# Defining the rules of engagement: B cells, antibodies and cancer control

**DOI:** 10.1038/s41423-026-01422-x

**Published:** 2026-05-06

**Authors:** Stephen L. Nutt, Julie Tellier, Jessica Da Gama Duarte

**Affiliations:** 1 The Walter and Eliza Hall Institute of Medical Research, Parkville, VIC Australia; 2 Department of Medical Biology, University of Melbourne, Parkville, VIC Australia; 3 Department of Cancer Medicine, School of Translational Medicine, Monash University, Melbourne, VIC Australia; 4 Olivia Newton-John Cancer Research Institute, and La Trobe School of Cancer Medicine, Heidelberg, VIC Australia

**Keywords:** Humoral immunity, Tumor-infiltrating B cell, Plasma cell, Immunotherapy, Tertiary lymphoid structure, Tumor-specific antibody, Tumour immunology, Tumour immunology, Antibodies

## Abstract

Immunotherapy has emerged as one of the mainstays of cancer therapy. To date, immunotherapy research has focused heavily on approaches to modulate the anti-tumor activities of T cells, with other immune components of the tumor microenvironment, including B cells, receiving considerably less attention. Mounting evidence has shown that B cells, plasma cells, and the antibodies they produce can impact tumor control. B cells can have both anti-tumor activity, particularly when organized into tertiary lymphoid structures, and pro-tumorigenic roles in some settings. The rules underlying the complex interplay between B cells, other components of the tumor microenvironment, and the cancer cells themselves are only now being elucidated, but anti-tumor activity appears to be associated with distinct B-cell subpopulations and differentiation trajectories, as well as depending on the class of antibodies produced. Thus, the differentiation state of tumor-infiltrating B cells and the quality of antibodies produced may serve as prognostic markers of favorable patient outcomes. This review focuses on recent research that highlights how B-cell heterogeneity influences anti-cancer immunity and how this knowledge could be harnessed to develop B-cell-based immunotherapies and to fully utilize the power of antibody-based cancer diagnosis and patient stratification.

## Introduction

It is well known that the frequency of immune cell infiltration in solid cancers is correlated with improved prognosis and responsiveness to immunotherapies, with “hot” tumors being associated with the presence of many immune cell types, while this infiltration is lacking in “cold” tumors [1, 2]. Immune infiltrates include both the innate (macrophage, neutrophil, dendritic cell and natural killer (NK) cell) and adaptive (T and B cell) arms of the immune response. Beyond the simple frequency of immune cells in the tumor microenvironment (TME), the organization of infiltrating cells into tertiary lymphoid structures (TLSs) is also critical, as the presence, anatomical maturity, and cellular interactions of these structures generally indicate a better patient prognosis [3–5].

To date, attention in the tumor immunity field has focused predominantly on T cells. Tumor-infiltrating CD8^+^ T cells can directly kill tumor cells through their cytotoxic activity, while CD4^+^ T cells are more heterogeneous in their activity, with subsets specialized for immune modulation, including both the promotion and suppression of anti-tumor immunity. Although activated T cells are highly effective at controlling tumors, their chronic stimulation leads to impaired functionality, a process termed T-cell exhaustion, which is associated with the increased expression of a series of negative regulatory “immune checkpoint” proteins on the cell surface that can interact with ligands expressed on both immune cells and the cancer cells themselves [3]. The therapeutic blockade of these checkpoint proteins or their ligands provides the basis for most current immunotherapy protocols [6].

In addition to direct anti-tumor activity, a major function of T cells is to promote B-cell responses and antibody production. This task is carried out by a subset of CD4^+^ T cells termed T follicular helper (Tfh) cells, which reside in the lymphoid follicles of secondary lymphoid organs (SLO) and in TLSs in the context of cancer [7]. In both SLOs and TLSs, the number of B cells typically vastly exceeds the number of Tfh cells. Consequently, B-cell infiltration is a canonical feature of “hot” tumors. Plasma cells (PCs), the terminal antibody-secreting stage of the B-cell lineage, are also a common feature of “hot” tumors, although the homing properties of PCs differ from those of B cells, and they are located predominantly outside TLSs [8]. Notably, in some tumor settings, PCs and the antibodies they produce have been found to be protective of the tumor [9–14]. Gaining an understanding of the rules underlying when B cells and PCs are beneficial for tumor control, compared with situations and possibly cancer types where humoral immunity is problematic, is a major goal for the field, as without this insight, it remains impossible to rationally unlock the power of humoral immunity to better treat cancer patients.

In this review, we discuss recent advances in the analysis of human tumor tissues, particularly with a focus on single-cell genomic approaches and high-parameter spatial techniques that have greatly refined our understanding of the function of B cells and PCs in the TME [15–17]. These approaches emphasize not only the presence or absence of B cells as a prognostic marker of patient outcome but also the differentiation state and trajectory of the B-cell response. These approaches also hold promise for identifying new B-cell-centric immunotherapy strategies that may offer orthogonal approaches to treat cancer and harness antibodies for improved diagnosis and treatment stratification of cancer patients.

## B-cell development and function

B cells develop in the bone marrow, where they undergo sequential rearrangement of their immunoglobulin heavy and light chains to produce cells that each carry a unique B-cell receptor (BCR) [18]. The BCR consists of two identical heavy chains and two light chains and includes an N-terminal variable region produced by immunoglobulin rearrangement that is the antigen-binding region. Success in this process generates immature B cells that express the membrane form of IgM and a differential splice variant called IgD. Immature B cells egress from the bone marrow and travel to SLOs, where they mature into follicular B cells. B-cell development can also generate more innate-like variants, including B1 cells that predominate in fetal life and in the peritoneal and pleural cavities, at least in mice, and marginal zone B cells that surround the lymphoid follicles of the spleen [19].

BCRs can detect a virtually infinite variety of possible targets, including both protein and nonproteinaceous antigens, across a very broad affinity range. Upon encountering their cognate antigen, which may include unique tumor antigens, B cells are induced to undergo rounds of rapid proliferation, known as clonal expansion. These activated B cells can undergo immunoglobulin class switch recombination (CSR), a process that excises the DNA sequences downstream of the heavy chain variable regions and ligates this sequence to a downstream alternative constant region to produce the IgG, IgA, and IgE isotypes. Activated B cells then either differentiate into short-lived antibody-secreting cells termed plasmablasts, or can continue proliferating in a defined area of the follicle, resulting in the formation of a transient structure called the germinal center (GC) [20]. GCs are sites of somatic mutation of the BCR, followed by selection and further proliferation of GC B cells harboring clones with increased affinity for their cognate antigen. The outputs of the GC are either memory B cells [21], which in many ways resemble the starting follicular B cells, with the exception of the increased affinity of their BCR for antigen, and antibody-secreting PCs.

Memory B cells can carry either an IgM or an IgG form of the BCR and are generally located in circulation. One subset of memory B cells that has received substantial recent interest in the context of aging, autoimmunity, and cancer immunity is atypical memory (Atm) B cells [22–24]. These cells are also known as age-associated B cells (ABCs) or CD27^−^IgD^−^ double negative (DN) cells and rely on the transcription factor ZEB2 [25, 26] and express the cell surface protein CD11c. Atm B cells are thought to be the consequence of chronic activation, have a reduced capacity to differentiate into antibody-secreting cells, and display signs of cellular exhaustion. As outlined below, several large-scale analyses of the TME have implicated Atm B cells in anti-tumor immunity.

PCs are the key source of antibodies in the body and can be extremely long-lived, with postmitotic PCs known to survive many decades in humans [20, 27]. Bone marrow is often thought of as the canonical organ that houses long-lived PCs; however, long-lived PCs are found in most, if not all, nonlymphoid tissues [28]. Compared with B cells, PCs tend to be located surrounding TLSs. PCs can produce tumor-specific and tumor-associated antibodies and have been proposed to have both pro- and anti-tumor effects, which may depend on the cancer type and the antibody isotype secreted.

## Uncertain role of B cells in cancer: historical data

Compared with corresponding healthy tissue, tumor-affected tissue generally contains more B cells and PCs. This observation has led to numerous studies evaluating the frequency and, in some cases, the phenotypic nature of tumor-infiltrating B cells. These studies initially took the form of immunohistochemistry but more recently included techniques such as high-dimensional flow cytometry and bulk and increasingly single-cell and spatial transcriptomics. While these approaches focus on the cells themselves, as outlined later in the review, other researchers have investigated the diagnostic and prognostic values of the antibody products of the humoral immune system.

Until recently, most studies that have investigated the prognostic value of tumor-infiltrating B cells and PCs have focused on cohorts of patients with a single defined cancer type. Although many of these studies concluded that the presence of B cells and, in particular, well-formed TLSs was associated with a favorable prognosis, other studies reported neutral and even negative effects of B cells and PCs (reviewed in [10, 13]). Numerous studies have also correlated patient responses to immunotherapy, including single agent or combination regimens using anti-PD-1, anti-PD-L1 and anti-CTLA-4 therapies, with the prevalence of tumor-infiltrating B cells (reviewed in [29, 30]). The presence of B cells has been shown to be of favorable prognostic value in numerous malignancies, including breast [31–33], liver [34, 35], colorectal and bladder [36] cancers, squamous cell carcinoma [37], sarcoma [38] and melanoma [39–41]. The correlations with PCs are more complex, with their prevalence being favorable in some studies of colorectal [42], renal [8], esophageal [43, 44] gastric [43] and lung [45, 46] cancers but unfavorable in others focusing on breast [47], gastric [48], liver [12], prostate [11], pancreatic [49] and ovarian [50] cancers, as well as melanoma [51].

## B-cell subsets in tumors

### Is the immunoglobulin isotype important?

One key variable in the humoral immune response to cancer is the class of antibodies produced. As outlined above, naïve B cells express the cell membrane-associated form of the IgM isotype. Although B cells can differentiate into PCs that secrete IgM, most tumor-specific B cells, particularly those generated in the GCs of lymph nodes and TLSs, undergo CSR to a downstream immunoglobulin isotype, predominantly IgG or IgA. The ‘choice’ of the isotype is determined by the signals present in the milieu in which the B cells are activated.

**Fig. 1:**
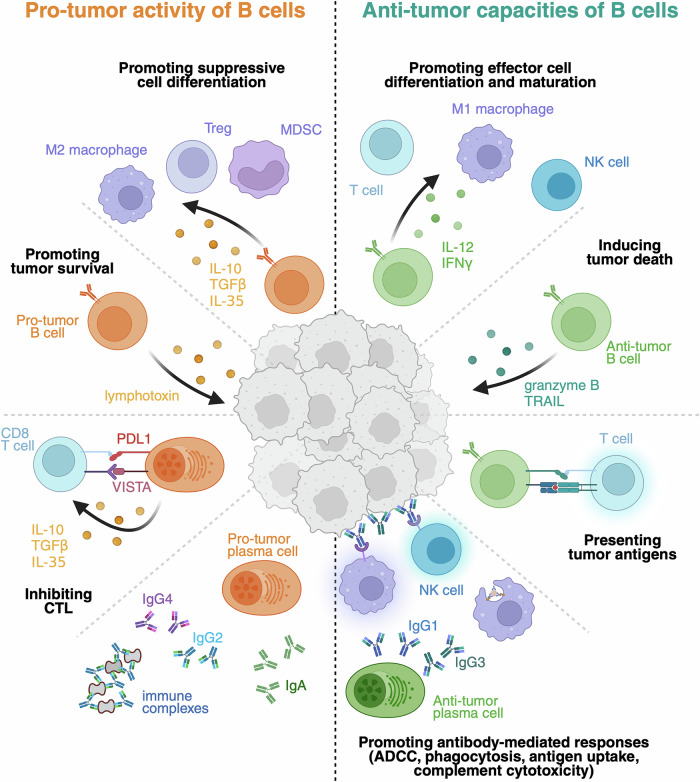
Multifaceted roles of B cells in tumors. On the left are the activities through which B cells favor tumor development, either by secreting pro-tumor factors, such as lymphotoxin [11, 12, 143], or by suppressing the immune response through the expression of inhibitory molecules (IL-10, TGFβ, IL-35, PDL1, and VISTA) [144]. The B-cell effector functions that are integral to the anti-tumor immune response, including the presentation of antigens to T cells, the secretion of proinflammatory cytokines (IL-12 and IFNγ), and direct cytotoxic capacities, are shown on the right [35]. The role of antibodies in tumor control is dependent on their isotype. IgG1 and IgG3 can efficiently bind to Fcγ receptors and mediate phagocytosis and cytotoxicity (Antibody-dependent cellular cytotoxicity and complement), whereas IgA and IgG4 lack these functions

The IgG1 subclass is the canonical anti-tumor antibody type, as this class can bind to the Fcγ receptors on myeloid and NK cells and thus trigger a process termed antibody-dependent cellular cytotoxicity (ADCC) and indirectly activate cytotoxic CD8^+^ T cells (Fig. 1). In contrast, the IgG2 and IgG4 subclasses are generally considered to indicate unfavorable responses. IgA is the most abundant antibody class in mucosal sites, including the gastrointestinal tract and female genital tract. Consequently, IgA PCs and antibodies predominate in colorectal [42], bladder and ovarian [14] cancers but can also accumulate in prostate cancer [11] and melanoma [50, 52]. IgA binds the PIGR receptor on epithelial cells and is transcytosed through these cells and secreted into the lumen of the gut to maintain homeostasis with the gut microbiome [53]. IgA does not trigger ADCC but can act as a neutralizing antibody without inducing an inflammatory response. Interestingly, in the context of ovarian cancer, IgA is bound to PIGR on the surface of cancer cells, and the transcytosis process allows anti-tumor IgA to bind to intracellular oncogenes and antagonize their function, rendering tumor cells susceptible to T-cell death [54, 55]. While the above example highlights an anti-tumor function for IgA, IgA PCs have been shown to have a negative prognostic value in hepatocellular carcinoma [12], melanoma [51], and advanced prostate cancer [11]. In these settings, the IgA PCs themselves contribute to a pro-tumor microenvironment through the high expression of immunosuppressive molecules, including IL-10, TGFβ, and IL-35, as well as the immune checkpoint molecule PD-L1 (Fig. 1). The secretion of TGFβ can create a self-sustaining loop between IgA PCs and regulatory T cells that promotes each other’s generation [56]. In addition, repertoire analyses in melanoma revealed that the abundance of IgA was associated with low clonality, which suggests that these antibodies are less specific than their IgG1 counterparts [57]. Like IgG4, IgA can form immune complexes with tumor and non-tumor antigens that favor inflammation and a suppressive phenotype in myeloid cells. In summary, while a high proportion of IgA is generally indicative of an immunosuppressive TME, these antibodies can play anti-tumoral roles in some specific tissue contexts, including ovarian cancer.

### Tumor-infiltrating memory B-cell subsets

Beyond their role as effector cells, B cells also actively contribute to the initiation and maturation of TLSs through the production of key lymphoid-organizing signals. In particular, the expression of lymphotoxin (LTα1β2) on activated B cells is required to initiate the activation of follicular dendritic cells, a crucial step of the GC response [58]. Notably, while lymphotoxin signaling is essential for TLS formation, it can also promote tumor growth, highlighting its context-dependent role within the TME [59]. CXCL13, previously known as B-cell-attracting chemokine 1, is produced primarily by follicular dendritic cells in B-cell follicles to recruit CXCR5^+^ B cells and Tfh cells, thereby promoting follicular organization and GC formation [60]. Together, lymphotoxin and CXCL13 signaling, along with other inflammatory mediators, contribute to a positive feedback loop that is critical for TLS development and maturation [61]. Importantly, these pathways can potentially be therapeutically targeted, with several preclinical studies investigating strategies to induce TLS formation and enhance anti-tumor immunity [62].

**Fig. 2:**
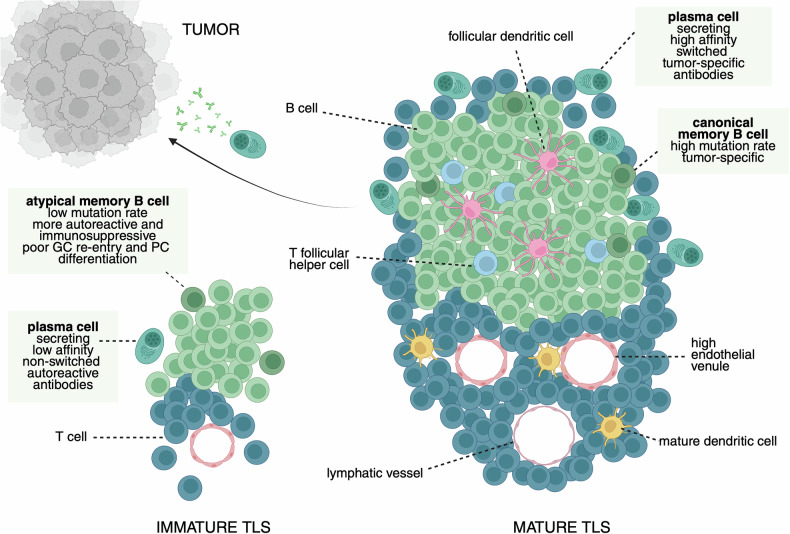
Tertiary lymphoid structure maturity and outputs in anti-tumor immunity. Schematic depiction presenting the divergent outputs (from a B-cell perspective) of TLSs depending on their maturation status. Immature TLSs are inefficient participants in the GC reaction, lack follicular dendritic cell networks in defined B-cell zones and mature dendritic cells in defined T-cell zones, and are associated with atypical memory B cells that are enriched in autoreactive cells. Atypical memory B cells and immature TLSs produce few plasma cells that secrete low-affinity unswitched antibodies. In contrast, mature TLSs, characterized by well-defined B- (CD19^+^CD20^+^) and T-cell (CD3^+^ and CD4^+^ or CD8^+^) zones, including high endothelial venules (PNAd^+^) and lymphatics, and the presence of mature dendritic cells (DC-LAMP^+^) and follicular dendritic cells (CD21^+^), are associated with canonical memory B cells with somatically mutated tumor-specific and tumor-associated BCRs [145]. Mature TLSs generate high-affinity classes of switched tumor-specific and tumor-associated antibodies through the efficient differentiation of plasma cells that migrate to the tumor bed

Not unexpectedly, TLSs are strongly enriched in B cells with an activated and/or memory phenotype, which is indicative of an engaged immune response [16, 17, 37, 63, 64]. Memory B cells are classically defined as CD19^+^CD38^−^CD27^+^IgD^−^ B cells and can be divided into IgM^+^ (unswitched) and IgM^−^ (switched) subsets. Switched memory B cells typically show somatic hypermutation (SHM) of their BCR as an indicator of GC selection. Despite not having undergone CSR, most IgM^+^ memory cells are also thought to have undergone a GC reaction, as they also show signs of SHM. Both memory subsets can either rapidly differentiate into plasmablasts or PCs or re-enter the GC to undergo more clonal expansion and affinity-based selection [21]. Both memory subsets are known to be abundant in TLSs (Fig. 2). More recently, Atm B cells expressing markers such as CD11c, FCRL4/5, and the transcription factor T-bet have been reported in TLSs in a cancer context [16, 17], including in non-small cell lung cancer [65, 66], breast cancer [67], and ovarian cancer [68]. The evidence supporting the relevance of Atm B cells in the TME is discussed below.

### B_regs_ and cancer

Regulatory B cells (B_regs_) represent another subset of B cells that have been suggested to be key players in anti-tumor immunity [69]. B_regs_ remain relatively poorly characterized, as they lack a uniform molecular definition beyond a high propensity to secrete immunosuppressive cytokines. Although the most extensively studied product of B_regs_ is IL-10, the production of IL-35 and TGFβ by Bregs has also been reported, and it is unclear whether these cytokines are produced by the same B_reg_ cells; thus, the extent to which different functionally distinct subpopulations of B_regs_ exist is unknown. Recently, a population of B_regs_ expressing the inhibitory checkpoint receptor VISTA correlated with tumor recurrence in non-small cell lung cancer [70]. CD19^+^CD20^+^ B cells with an immunosuppressive phenotype have also been reported in the tumor bed of intrahepatic cholangiocarcinoma biopsies [71]. It is, however, clear that many stages of B-cell differentiation can result in the production of suppressive cytokines, suggesting that these cells represent a functional state and not a distinct lymphoid cell type [69]. Indeed, some of the activities attributed to B_regs_ are attributed to IL-10-expressing plasmablasts and PCs [72, 73]. More research is needed to fully define both the nature and the function of these rare cells in cancer immunology.

## Tumor-infiltrating B cells in the OMICs era

The studies outlined above suggest that B cells and PCs can have both pro- and anti-tumorigenic properties depending on factors such as tumor type, therapies being utilized and potentially personalized features of the host immune system, and tumor progression (Fig. 1). While numerous studies have used whole-tumor genomic DNA or bulk RNAseq to quantify tumor-infiltrating B cells and PCs [9, 74, 75], these approaches lack the precision required to resolve the subtleties of B-cell subsets that may have diverse functions. To better define the importance of humoral immunity for anti-tumor immunity, several groups have constructed extensive pan-cancer atlases of tumor-infiltrating B cells and PCs [15–17]. These atlases provide single-cell transcriptome, chromatin accessibility, and BCR sequence information for up to 20 cancer types and several hundred patients, resulting in databases of more than 500,000 tumor-infiltrating B cells. The data are then supported by spatial information at the protein and transcriptome levels, as well as correlations with patient outcome and response to immunotherapy. It is hoped that the breadth of these approaches will allow a better definition of the rules of engagement of B cells and PCs in the TME than has been possible with studies that focus on a single cancer type.

These atlases clearly define the transcriptional diversity of the B-cell response. Ma et al. [16] reported that multiple activated and memory B-cell subsets, including Atm B cells, are enriched in the TME. Analysis of BCR clonality combined with single-cell transcriptome and chromatin accessibility profiling revealed the presence of two trajectories. In some tumors, B cells pass through a GC pathway, generating highly mutated and immunoglobulin class-switched PCs. This pathway is predominant in colorectal, gallbladder, lung, and stomach cancers, among others, and is often associated with IgG^+^ and IgA^+^ cells [16]. In contrast, other tumor types and individual patients carried tumors with a more prominent signature of an extrafollicular B-cell differentiation response characterized by low rates of CSR and SHM. The extrafollicular pathway was more prevalent in liver, pancreatic, breast, cervical, and head and neck cancers. The authors proposed a model in which Atm B cells are the primary progenitors for the extrafollicular response in the TME [16]. The extrafollicular pathway utilizes separate transcriptional regulators from the canonical GC pathway, including T-bet, and ultimately produces distinctly programmed GC-derived and extrafollicular-derived IgM^+^ PCs. In support of their proposed extrafollicular origin, compared with conventional memory B cells, Atm B cells in the TME were relatively less likely to have undergone CSR, had lower SHM, and were generally impaired in their ability to differentiate into antibody-secreting cells in vitro. Compared with memory B cells, Atm B cells also secrete immunosuppressive cytokines such as IL-10 and TGFβ and express more PD-L1. Analysis of the BCR repertoires of tumor-infiltrating B cells provided independent evidence of the developmental separation of the Atm B-cell-associated extrafollicular response and the more conventional GC response.

Mapping the anatomical location of B-cell populations in the TME revealed that both Atm and GC B cells are associated with a TLS signature, an expected finding given that TLSs are largely composed of B cells (Fig. 2). However, stratifying the TLSs observed in liver and colorectal cancer by maturation status revealed that Atm B cells were enriched in immature TLSs, whereas conventional memory B cells predominated in the most mature TLSs. In individual patient biopsies, as TLSs mature, Atm B cells move from the center of the structure toward the periphery and are replaced by more follicular-like B-cell subsets. Thus, GC B-cell-dominated cancers were enriched in the most mature TLSs, which are structurally organized to support B-cell clonal expansion, CSR and antibody production. PCs, in contrast, were always associated with the tumor bed and were physically removed from the TLS [15, 16]. While the presence of mature TLSs is correlated with robust anti-tumor B-cell responses, deciphering to what extent TLS maturation is a driver or a consequence of effective anti-tumor immunity remains key.

This clear segregation of the extrafollicular and GC pathways in the TME raises the question of the processes responsible for these divergent trajectories. Ma et al. [16] investigated the metabolic status of memory and Atm B cells using computational analysis of single-cell transcriptome data and mass spectrometry-based metabolomics and reported that glutamine levels were higher in extrafollicular malignancies such as liver and kidney cancer than in GC-dominant cancers of the gastrointestinal tract. The results of cell culture experiments revealed that the addition of glutamine promoted the differentiation of naïve B cells from healthy individuals toward an Atm B-cell fate while decreasing the number of memory B cells. These experiments suggest that altered glutamine in the TME may directly impact the productivity of the humoral immune response. Such approaches, if confirmed in more physiological settings, such as mouse tumor models, may suggest strategies to manipulate the humoral immune response as a novel cancer immunotherapy approach.

A second pancancer B-cell atlas of similar scale and complexity was also recently reported by Yang et al. [17]. They also a large variation in the frequency of tumor-infiltrating B cells, ranging from extremely few in uveal melanoma and squamous cell carcinoma to frequent (20–50% of all CD45^+^ cells) in colorectal, stomach, and esophageal cancers. They also reported stark differences in the frequency of tumor PCs, with PCs being most abundant in liver, colorectal, and stomach cancers. Notably, however, there was large interpatient variability in the frequency of PCs. For example, the PC frequencies in individual breast cancer samples ranged from <5% to >95% of all B cells. Analysis of the tumor PCs revealed that IgG1 was the most frequent immunoglobulin isotype, including in tumors from the gastrointestinal tract where IgA PCs are abundant in normal tissue, suggesting a tumor-specific immune response. Interestingly, there was also evidence of shared BCR clonality with upstream isotypes such as IgM, suggesting CSR within the TME.

Yang et al. [17] also reported that Atm B cells (referred to as ABCs in that study) were present in all cancer types, although again, considerable variation in abundance was detected between individual patient samples. These findings were supported by those of Fitzsimons et al. [15], who developed a B-cell atlas from published data across 7 cancer types.

The abovementioned pan-cancer atlas studies also examined the prognostic value of measuring B-cell diversity in the TME. Ma et al. [16] used multiple independent cohorts of colorectal, stomach, lung and liver cancer patients to demonstrate that Atm B cells are correlated with poor survival outcomes, whereas in melanoma and liver cancer, Atm B cells are correlated with resistance to anti-PD-1-based immunotherapy. In contrast, Yang et al. [17] used data from The Cancer Genome Atlas (TCGA) to show that the prevalence of Atm B cells correlated with prolonged pan-cancer survival, which is in agreement with the findings of studies in lung [64] and liver [35] cancers. Yang et al. instead reported that a memory B-cell population with a transcriptional signature of a stress response was linked to poor patient survival. Previous similar studies have also reported stress response signatures in cancer-associated NK cells [76] and T cells [77], suggesting a general mechanism in the TME. The exact reason for these different conclusions remains unclear; however, Ma et al. [16] and Fitzsimons et al. [15] treated Atm B cells as a single population, whereas Yang et al. [17] separated these cells into 2 distinct subpopulations, with only the FCRL4^+^ Atm B cells being relevant in the TME, an experimental difference that may impact the conclusions of each study. Notably, the origins of Atm B cells remain unclear. Ma et al. [16] suggested that these cells were derived from an extrafollicular immune response, whereas Yang et al. [17] provided evidence for a GC origin. There is emerging evidence in models of infection and autoimmunity that Atm B cells can be derived from either pathway, suggesting that the balance of extrafollicular versus GC-derived Atm B cells may also vary in the context of cancer [24–26]. Future reanalyses of the gene expression signatures derived from these datasets using the same patient cohorts, such as a matched TCGA dataset, should help clarify these important points.

## Functions of tumor-infiltrating B cells

Tumor-infiltrating B-cell subsets can contribute to the anti-cancer immune response through several distinct pathways, including antigen presentation and costimulatory functions, as well as the secretion of antibodies and immune-modulatory cytokines (Fig. 1). Activated B cells can process and present tumor antigens to CD4^+^ T cells via the MHC class II pathway. This process promotes both direct Tfh development and function in the GC and indirectly supports CD8^+^ T-cell function, potentially via IL-21 expression [78]. It is difficult to disentangle the anti-tumor functions of B-cell antigen presentation from the direct impact of the secretion of tumor-specific and tumor-associated antibodies in clinical samples. However, studies in mice harboring a B-cell-specific inactivation of Blimp1 that cannot produce PCs or secrete antibodies have revealed that the prolonged antigen presentation and GC reaction that accompanies a block in PC differentiation resulted in clearly enhanced anti-tumor immunity [79].

A major pathway through which humoral immune cells influence tumor growth is through the secretion of tumor-specific and tumor-associated antibodies. As discussed in the next section, these antibodies are frequently present in cancer patients, and their presence can correlate with that of autoantibodies, such as those observed in autoimmune conditions. In some circumstances, these antibodies can be mapped to defined foreign antigens, such as in the case of human papilloma virus (HPV)-associated cancers [80], although in many cancer settings, the nature of the neoantigens remains unclear. A study in ovarian cancer revealed that patient biopsies were frequently coated with antibodies, and extensive molecular profiling of the origins of these antibodies revealed two classes of B-cell clones [81]. In the first situation, the BCR sequences were germline (lacking SHM) and encoded autoreactive antibodies, whereas the second type of tumor-reactive antibody was produced from B-cell clones where the BCR lacked any tumor reactivity in its germline configuration and instead acquired this activity through somatic mutation and selection in the GC. Thus, tumor-specific antibodies can evolve from nonreactive B-cell precursors. Consistent with these findings, GC reactions within mature TLSs in pancreatic cancer correlate with predicted neoantigen expression and enhanced humoral immunity, supporting a role for neoantigen-driven selection in shaping B-cell maturation and antibody responses in human tumors [82].

## The value of tumor-specific and tumor-associated antibodies in oncology

Antibodies secreted against T-cell-dependent tumor antigens can be used as surrogate markers for the presence and extent of an adaptive immune response, as these depend on effective immune recognition of solid tumors [83]. The antibody repertoire also reflects the dynamics of an evolving immune response, including changes in tumor antigen expression, the TME, treatment-related effects, anti- or pro-tumoral activity, and immune memory. As outlined above, whether antibodies contribute to anti- or pro-tumor immunity depends on the class and subclass of antibodies produced, with IgG1 and IgG3 thought to be favorable and IgG2 and IgG4 unfavorable [84]. IgA, on the other hand, has been proven to be complex, with reported anti- and pro-tumoral roles in different cancers [85]. Moreover, while T-cell-dependent antigens can lead to the secretion of cognate high-affinity IgG and IgA, they can also lead to transient modest-affinity IgM [25]. Antibody responses are therefore diverse and polyfunctional and can have distinct clinical implications [86].

### Measuring antibodies in cancer

**Fig. 3:**
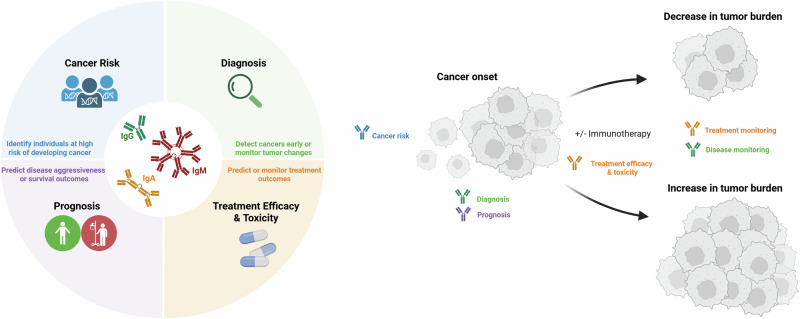
Tumor-specific and tumor-associated antibodies are used as cancer biomarkers. On the left is a diagram depicting the different clinical uses of antibody profiling in oncology, including IgM, IgG, and IgA antibodies, to determine cancer risk, diagnosis, prognosis and treatment outcomes. On the right is a representation of the clinical timeline during which these measurements can occur

Owing to their ease of extraction via blood draw, long half-life, production in premalignant or early-stage disease, presence across disease stages and cancer types and systemic representation, circulating antibodies against tumor antigens are attractive cancer biomarkers [87]. Unlike invasive tissue-based biomarker approaches, the circulating antibody repertoire can also overcome tumor heterogeneity concerns, both within tumors and across tumors. Blood-based biomarkers also enable multiple measurements to be performed during the patient’s cancer progression, including determination of cancer risk; at diagnosis, progression, and recurrence; and during treatment monitoring (Fig. 3). However, while some cancers are known to be highly immunogenic, particularly those with high somatic mutation frequencies, such as melanoma [88], this is not the case across all cancers. Hence, the applicability of antibody profiling may vary across cancer types.

Tumor-specific antibodies recognize unique antigens not found in normal cells that usually result from point mutations in broadly expressed genes, whereas tumor-associated antibodies recognize antigens similar to those found in normal cells but are either modified or produced in greater quantities [89]. Antibodies against known tumor-specific antigens are notably preferred when ideal biomarkers are considered, particularly those termed neoantigens, as these are specific to cancer, although those against tumor-associated antigens (autoantibodies) also hold value [90, 91]. Moreover, when discovery-based antibody profiling is performed to identify potential cancer biomarkers, detected antibodies can also inform novel tumor antigen-based therapeutics, including cancer vaccines [92] and adoptive cell therapies [93].

While most of the literature is focused on the antibody repertoire in circulation, elucidating the local antibody repertoire may also be valuable. The latter encompasses tumor-draining lymph nodes (tdLNs) and tumors associated with TLSs [8]. Investigating the local antibody repertoire requires the isolation of antibody-secreting cells from tdLNs [94, 95] or tumors [96, 97] and their subsequent ex vivo supplemented culture, where they continue to secrete antibodies for several days. The culture supernatant can then be used for downstream antibody profiling to determine antigen specificity and correlations to the systemic antibody repertoire. Although the local antibody repertoire may be enriched for tumor-associated antigens or neoantigens, reflecting ongoing antigen-driven selection within TLSs, this may not be fully captured by circulating antibodies, indicating that intratumoral and systemic humoral immunity may arise and be maintained in distinct anatomical compartments (reviewed in [98]).

Antibody profiling is typically performed using established methods, including planar-based microtiter or slide assays, such as enzyme-linked immunosorbent assays (ELISAs), high-sensitivity ELISAs, and protein microarrays, as well as suspension-based assays, such as multiplex bead immunoassays (e.g., Luminex). Standard ELISAs typically achieve detection limits in the low pg/mL range, whereas high-sensitivity ELISAs can reach subpg/mL concentrations, enabling the detection of low-abundance antibodies often present in early-stage disease [99]. Protein microarrays offer comparable or, in some cases, enhanced sensitivity (platform-dependent) and provide the highest multiplexing capacity, enabling simultaneous profiling of hundreds to thousands of antigens [100, 101]. Luminex bead-based assays combine high sensitivity, often comparable to ELISA, with moderate multiplexing capacity (~10–100 analytes per assay), offering a flexible alternative for medium-scale panels [100, 102]. Therefore, the antibody profiling method used depends on the research question, which needs to consider assay sensitivity and multiplexing capacity (Supplementary Table 1). To ensure specificity, antigen content should be tailored to the study context; for example, panels focusing exclusively on tumor-associated antigens are most appropriate in cancer biomarker studies, reducing interference from autoantibodies present in healthy individuals or patients with autoimmune conditions. In addition, the platforms themselves are designed to minimize non-specific binding through optimized blocking buffers, wash steps, and surface chemistries, which help reduce background signals and improve assay specificity. When low antibody titers, which commonly occur with early-stage disease or low tumor burden, are being measured, assay sensitivity is critical. Similarly, when screening a large number of antigens or patient samples, multiplexing capacity is also an important consideration. Given that most B-cell epitopes are discontinuous in sequence, protein microarrays with correctly folded full-length proteins, rather than peptide arrays with linear continuous epitopes, are critical for investigating antibodies against conformational epitopes [103].

Antibody profiling methods usually require prior knowledge of antigens of interest, as screens are limited to the content of the immobilized antigen. Antigen discovery across cancer types has thus been largely explored over the years, with different methods used across researchers, including serologic expression cloning (SEREX), western blotting, mass spectrometry, and phage display [90, 104].

The application of antibody profiling to identify candidate cancer biomarkers requires the use of robust statistical methods and the incorporation of adequate prediction algorithms that enable successful validation for clinical implementation. These include the use of adequately powered training cohorts to construct a predictive algorithm and independent validation cohorts [105, 106]. Studies have also previously confirmed antibody profiling data by investigating cognate antigen expression in matched tumor tissues using cancer-specific tissue microarrays [107] or public protein expression data (e.g., The Human Protein Atlas [108]) obtained using immunohistochemistry.

### Cancer risk

As indicated above, circulating antibodies can precede cancer onset and be present in premalignant disease. Hence, several studies have investigated the value of antibodies in predicting an individual’s risk of developing cancer (Fig. 3). A recent systematic review including 273 articles summarized associations between infection-, tumor-, or self-related autoantibodies and site-specific cancer risk in the general population or specific target populations [109]. With respect to infection-related antibodies, IgG, IgM, and IgA antibodies against EBNA and VCA (EBV exposure) are associated with the development of nasopharyngeal carcinoma; IgG antibodies against HPV6, HPV16, and HPV18 (HPV exposure) are associated with oropharyngeal, cervical, and anogenital cancers, respectively; and IgG and IgM antibodies against HBs and HBc (Hepatitis B virus exposure) are associated with hepatocellular carcinoma and pancreatic cancer; IgM antibodies against HCV (Hepatitis C virus exposure) are associated with hepatocellular carcinoma; IgG antibodies against *Chlamydia trachomatis* are associated with ovarian cancer; and IgG and IgA antibodies against CagA (*Helicobacter pylori* exposure) are associated with gastric cancer. For tumor- and self-reactive antibodies against single antigens or a panel of antigens, there is also evidence of positive associations with cancer risk. Among these, antibodies against p53 are commonly associated with the subsequent development of cancers, particularly for breast, colorectal, esophageal, lung, ovarian, and endometrial cancers. These antibodies are thought to result from alterations or mutations in immunogenic *TP53* [110]. This highlights the value of screening specific antibodies in target populations, including high-risk individuals (e.g., carriers of familial cancer risk genes) and those with suspicious or benign lesions (e.g., premalignant lung lesions or thyroid nodules). This systematic review also revealed associations between autoimmune disease and site-specific cancer risk, including celiac disease, autoimmune myopathies, scleroderma, and autoimmune thyroiditis [109]. Among these, autoimmune thyroiditis patients with antibodies against Tg and TPO are commonly associated with the subsequent development of thyroid cancer.

### Diagnosis

When detected early, cancers are often localized, and surgery alone may be curative. However, when detected late, cancers often progress and invade nearby organs, lymph nodes, and distant sites, thereby substantially increasing disease morbidity and the risk of cancer-related death. National cancer screening programs, including those for breast, cervical, bowel, and, more recently, lung cancer, aim to detect cancers early and often consider risk factors, including familial cancer risk genes or history [111]. Understandably, among the many uses of circulating antibodies as cancer biomarkers, diagnostics remain the most well explored, particularly because of their non-invasive ability to detect premalignant and early-stage cancers, at times even those preceding clinically apparent disease [112] (Fig. 3). In addition to the abovementioned methods required for adequate cancer biomarker studies, it is important to include adequate control cohorts. When cancer diagnostics are considered, age- and sex-matched healthy cohorts are needed to adequately identify candidate antibodies that are exclusively detected in cancer patients and, as such, are absent in healthy individuals. For candidate diagnostic biomarker discovery, these healthy cohorts should exclude individuals with a prior history of cancer and those with known autoimmune conditions, as these may introduce errors, albeit this warrants further consideration when testing identified antibody biomarkers in a real-world setting. Moreover, it is important to consider tissue-matched benign conditions and thereby the inclusion of a second control cohort that adequately represents a repertoire of common benign conditions likely present in the general population. This is particularly important in clinical settings where lesions are suspected of malignancy and are often monitored closely for changes over a specific timeframe prior to intervention, which is a common practice for identifying lung nodules.

Despite extensive research across cancer types, only one circulating antibody-based diagnostic test has been evaluated in a clinical trial setting [113] and commercialized, EarlyCDT® Lung. This test aims to aid in the early detection of lung cancer in high-risk individuals and provides an effective assessment of lung cancer risk in indeterminate pulmonary nodules (https://www.earlycdt.com/). It consists of an ELISA-based assay measuring circulating IgG antibodies against six tumor-associated antigens (p53, NY-ESO-1, CAGE, GBU4–5, Annexin 1, and SOX2) [114–116]. Initial studies revealed that up to 40% of lung cancers could be detected [117, 118], including early-stage disease. Several other promising antibody-based diagnostic biomarkers have been reported in the literature across solid tumors, particularly with the advent of methods that enable the interrogation of antibodies against the entire human proteome [119]. Notably, while antibody specificities can be shared between cancer types, unique signatures are often reported, suggesting the potential of a pan-cancer antibody-based diagnostic test capable of distinguishing individual cancer types. However, the clinical implementation of such tests should be limited to screening high-risk populations or those with suspected cancers to avoid the overdiagnosis and potential overtreatment of indolent cancers.

### Prognosis

The ability to infer prognosis is highly valuable and can inform personalized medical approaches for cancer patients. Specifically, patients who are likely to experience recurrence or metastasis may benefit from more aggressive treatment approaches, including adjuvant treatment after curative surgery, as well as more regular monitoring. On the other hand, patients deemed to have indolent cancers may be spared from potentially toxic and costly therapeutic interventions. Circulating antibodies can also be prognostic in cancer, with reported associations with tumor stage, overall survival, recurrence- or progression-free survival, nodal involvement, and distant metastases (Fig. 3, reviewed in [120]). Among these, p53 antibodies have been commonly reported to be prognostic in colon, lung, breast, and gastric cancers, with a meta-analysis including 12 articles reporting that high levels of circulating p53 antibodies correlated with poor survival outcomes [120]. This prognostic value is in part attributed to the genomic and proteomic expression of these antigens, which are often cancer type- and subtype-dependent and confer pro-tumorigenic features that contribute to tumor progression or spread [121, 122]. Moreover, antibody titers have been shown to reflect tumor burden; thus, longitudinal changes in the antibody repertoire can be indicative of disease progression or regression [123].

While most studies have focused on circulating antibodies, these antibodies can also be produced locally within tumors as products of TLSs [8, 84]. It is thus plausible that the prognostic value of TLSs may in part be attributed to their antibody output, particularly when considering antigen specificity and antibody isotypes or subclasses.

## Immunotherapy efficacy and toxicity

Autoimmunity can be referred to as beneficial at times, particularly when cancer prognosis is considered. Although autoimmune diseases can increase the risk of site-specific cancers (e.g., vitiligo and melanoma), these often correlate with favorable disease outcomes [124]. Similar treatment-induced phenomena have been reported in cancer patients who experience immune-related adverse events (irAEs) as a result of immunotherapy with checkpoint blockade.

Immunotherapies, particularly those targeting checkpoint molecules such as PD-1 and CTLA-4, aim to stimulate the immune system to overcome immune evasion [125]. irAEs are common immunotherapy-related side effects that mimic autoimmune conditions, with high-grade (grade 3 or 4) irAEs requiring immediate clinical intervention, often leading to treatment cessation, and can be life-threatening [126]. In addition to the above-described features, “hot” or immunologically active tumors likely to benefit from first-line immunotherapeutics have been defined as having preexisting robust antibody responses, highlighting their potential use as predictive cancer biomarkers [127]. It is thus likely that preexisting antibody responses may be indicative of overall “immune health” or “immune fitness”, predisposing patients to benefit from treatments targeting the immune system [128]. Indeed, antibody profiling can now be used to predict responses to checkpoint blockade across cancer types [129, 130].

Antibody production is also induced as a consequence of some cancer treatments, suggesting that antibodies can be used to monitor treatment responses or changes in tumor burden. Clinical trials using tumor antigen-based cancer vaccines often involve the detection of cognate antigen-specific antibody (and T cell) responses or epitope spreading to determine treatment efficacy (e.g., the NY-ESO-1 vaccine in melanoma [131]). Tumor cell death itself can result in the release of tumor antigens into circulation, including those that may not have been previously “seen” by the immune system, thereby triggering the production of antibodies [87]. Moreover, checkpoint blockade can directly or indirectly affect B cells and antibody production [132, 133], resulting in spikes in antibody titers or de novo antibody responses, which warrants consideration when assessing on-treatment antibody kinetics. However, whether these antibodies directly contribute to the anti-tumor response is still poorly understood.

Similarly, when patients have an “overactive” immune system or underlying autoimmune condition, it is plausible that excessive or dysregulated autoantibody production may predispose patients to develop irAEs when they are treated with checkpoint blockade. Indeed, several studies have also confirmed that preexisting or early on-treatment circulating autoantibodies can predict high-grade irAEs, preceding the clinical detection of these irAEs and at times even pinpointing the affected organ [120, 134, 135]. However, whether these antibodies are simply bystanders or pathologic remains unknown.

## Future directions

Rapid advances in genomic profiling, particularly at the single-cell level, have demonstrated that B cells and TLSs are generally associated with favorable clinical outcomes for cancer patients. The antibodies produced by tumor-specific and tumor-associated humoral immunity are valuable biomarkers of cancer diagnosis, responsiveness to treatments and patient prognosis. The recent realization that it is not necessarily the quantity of tumor-infiltrating B cells that is most important but instead the type of response and the differentiation trajectories employed that are key to an effective anti-tumor humoral immune response raises the possibility that immunotherapy strategies that either increase the most productive anti-tumor antibody responses or impede the suppressive pathways will be beneficial for cancer patients. However, to date, no B-cell-targeted strategies have entered clinical practice as immunotherapies for non-B lineage malignancies.

As outlined in this review, perhaps the greatest barrier to the successful application of B-cell-based immunotherapy is the need to develop and validate methods to stratify cancer types and individual patients on the basis of the functionality of tumor-infiltrating B cells; that is, whether B-cell activity needs to be depleted or enhanced to aid in tumor control. In this regard, the findings from the large pan-cancer studies reviewed here [15–17] provide a potential way forward, as they highlight the relevance of distinct B-cell trajectories, such as the pathway that generates Atm B cells as biomarkers of tumor responsiveness to therapy and thus patient prognosis.

In circumstances where B cells have a pro-tumor function, strategies such as antibody-mediated B-cell and/or PC depletion could be readily applied. These therapies are routinely used in the clinic to treat B-cell malignancies and autoimmune conditions [136, 137]. Although these therapies typically compromise the immune system of patients, this risk is well known and can be mitigated using immunoglobulin replacement therapy [137].

In the alternative scenario where B cells have anti-tumor function, strategies that further promote humoral immunity are needed. Novel treatments in development to increase B-cell responses include the use of agonistic anti-CD40 monoclonal antibodies [138] or toll-like receptor ligands [139]. B-cell epitope-based vaccines, usually in combination with T-cell epitopes, as well as antigen-specific B cells, are also currently in early-stage clinical trials [140]. One example of this approach is the adoptive transfer of EBV-transformed B-cell lines that have been engineered to express tumor neoantigens that are mutated in pancreatic cancer [141]. Finally, autoantibodies may have utility beyond prognostic markers, as one study showed that a lupus-specific mAb sensitized tumor cells to chemotherapy and radiation therapy [142].

Although it remains to be determined whether these B-cell-modulating approaches will be effective in treating non-B-cell malignancies, one key advantage of this approach is that it is largely independent of T-cell responses and thus is likely to function synergistically with established and near ubiquitous approaches, such as immune checkpoint inhibitors. Finally, there is undoubtedly great potential to better refine the strategies to identify and make use of tumor-specific or tumor-associated antibodies as highly sensitive biomarkers to assist in the diagnosis, monitoring, and treatment selection of patients across the whole spectrum of cancer types.

## Supplementary information

Supplementary table 1
