# Supplementary material for: Defining the rules of engagement: B cells, antibodies and cancer control
Source: Cell Mol Immunol. 2026 May 6;23(6):607–18. doi: 10.1038/s41423-026-01422-x (PMC13222348; doi:10.1038/s41423-026-01422-x)
Supplement: Supplementary file 1 — Supplementary table 1 [file 41423_2026_1422_MOESM1_ESM.docx]

**Supplementary Tables**

**Supplementary Table 1. Technical comparison of standard antibody detection methods.** The table summarizes the sensitivity, throughput, required sample volume, typical uses, and key limitations of standard ELISA, high-sensitivity ELISA, protein microarrays, and Luminex bead-based assays. The sensitivity ranges are platform-dependent and reflect typical detection limits for antibodies in serological assays. Throughput refers to the number of analytes that can be assessed per assay, and sample volume indicates approximate input requirements. This comparison is intended to guide researchers in selecting the most appropriate method on the basis of study scale, target abundance, and practical considerations such as infrastructure and cost.

| **Method** | **Sensitivity** | **Throughput** | **Sample Volume** | **Typical Use** | **Key Limitations** |
| --- | --- | --- | --- | --- | --- |
| **Standard ELISA** | Low-pg/mL range | Low | Moderate–High | Targeted validation; clinical assays | Low throughput; limited multiplexing |
| **High-sensitivity ELISA** | Subpg/mL range | Low | Low–Moderate | Detection of low-abundance antibodies | Low throughput; limited multiplexing; higher cost per analyte |
| **Protein Microarrays** | Sub- to low-pg/mL range (platform-dependent) | High | Low | Discovery-scale profiling (proteome- or immunome-wide) | Requires specialized infrastructure |
| **Luminex Bead-based assays** | Low-pg/mL range | Moderate | Low | Medium-scale profiling; validation panels | Panel design constraints; potential cross-reactivity |
